# MR-guided high-focused ultrasound for renal sympathetic denervation—a feasibility study in pigs

**DOI:** 10.1186/2050-5736-2-12

**Published:** 2014-08-04

**Authors:** Patrick Freyhardt, Lilian Heckmann, Alexander Beck, Nicola Stolzenburg, Jörg Schnorr, Julia Kamp, Jan L Rinnenthal, Bernd Hamm, Rolf W Günther, Florian Streitparth

**Affiliations:** 1 Department of Radiology, Charité - Universitätsmedizin Berlin, Augustenburger Platz 1, Berlin 13353, Germany; 2 Department of Neuropathology, Charité - Universitätsmedizin Berlin, Berlin 13353, Germany

**Keywords:** Hypertension, Renal sympathetic denervation, MRgHiFUS sympathicolysis

## Abstract

**Background:**

Renal sympathetic denervation has recently gained clinical relevance for the treatment of therapy-resistant hypertension. Denervation is currently mainly performed using catheter-based transarterial radiofrequency ablation of periarterial sympathetic nerve fibers. Since this approach has numerous limitations, we conducted a study to evaluate the feasibility, safety, and efficacy of magnetic resonance-guided high-focused ultrasound (MRgHiFUS) for renal sympathetic denervation in pigs as an alternative to catheter-based ablation.

**Methods:**

Renal periarterial MRgHiFUS was performed under general anesthesia in ten pigs. Blood pressure measurements and magnetic resonance imaging (MRI) of the kidneys, renal arteries, and surrounding structures were obtained immediately before and after the interventions and after 4 weeks. Histological examinations of periarterial tissues and determination of renal norepinephrine (NE) concentration were performed to assess treatment efficacy.

**Results and discussion:**

In each pig, 9.8 ± 2.6 sonications with a mean energy deposition of 2,670 ± 486 J were performed. The procedure was well tolerated by all pigs. No major complications occurred. MRgHiFUS induced periarterial edema in three pigs, but only one pig showed corresponding histological changes. The NE level of the treated kidney was lower in five pigs (-8% to -38%) compared to the untreated side. Overall, there was no significant difference between the NE values of both kidneys in any of the treated pigs. Postinterventional MRI indicated absorption of ultrasound energy at the transverse process and fascia.

**Conclusion:**

MRgHiFUS had some thermal periarterial effects but failed to induce renal denervation. Insufficient energy deposition is most likely attributable to a small acoustic window with beam path impediment in the porcine model. Since HiFUS treatment in humans is expected to be easier to perform due to better access to renal sympathetic nerves, further studies of this method are desirable to investigate the potential of MRgHiFUS as an alternative for patients not suitable for catheter-based renal sympathicolysis.

## Background

Hypertension is a major health burden and is on the rise, especially in industrialized countries. About half of the patients diagnosed with hypertension in the USA and 10% worldwide suffer from resistant hypertension, defined as a persistent elevation of blood pressure (BP) despite treatment at target dose with a combination of at least three antihypertensive drugs from complementary classes including a diuretic [1-3].

Renal sympathetic efferent and afferent nerves play an important role in the initiation and maintenance of resistant hypertension. Elimination of these nerve fibers by surgical resection of the splanchnic renal innervation was already introduced as a treatment for hypertension in the 1930s [4]. However, due to a high incidence of complications, the surgical method was abandoned in favor of medical treatment, which became increasingly available and effective.

With the technical advances of recent years, denervation of renal sympathetic nerve fibers using an intravascular radiofrequency ablation (RFA) catheter has become a promising method for lowering blood pressure in patients with refractory essential hypertension [5,6].

The results of the HTN-1 and HTN-2 trials [5,7] have spawned the development of multiple RFA systems including multielectrode RFA catheters and balloon-mounted RFA catheters with or without irrigation. Though, there exist drawbacks of catheter-based invasive ablation, e.g., not all patients are suitable for the catheter-based approach, as the renal arteries must have a certain diameter and length for catheter insertion [8]. The method is invasive and may cause complications related to arterial access and intravascular advancement of the RFA catheter. Furthermore, long-term data on possible RFA-related vascular wall injury as seen in a preclinical model [9] or hemodynamically relevant renal artery stenosis [10] are still lacking.

Apart from procedural aspects, clinical outcomes of treated patients are very variable. Finally, the latest results from the HTN-3 trial do not show a significant reduction of systolic blood pressure in patients with therapy-resistant hypertension 6 months after RFA-based renal artery denervation compared with a sham control [1]. Therefore, it is justified and necessary to investigate other potential methods for renal sympathetic denervation.

High-focused ultrasound (HiFUS) is a noninvasive method for tissue ablation that destroys tissue by directing a high-power ultrasound beam on a small focus. Within the focal volume, the resulting increase in temperature of up to 60°C–90°C leads to soft tissue damage sparing the surrounding tissue with a small boundary between damaged and spared tissue less than ten cells wide [11]. Magnetic resonance imaging (MRI) with its high intrinsic soft tissue contrast is ideal for visualizing the target structures and planning the procedure. At present, MRI-guided HiFUS (MRgHiFUS) is approved for the treatment of uterine fibroids and painful bone metastases in Europe and the USA. Experimental applications of HiFUS are currently investigated in numerous ongoing trials and range from the treatment of different tumor entities and occlusion of bleeding vessels to the ablation of arrhythmogenic cardiac tissue or enhancement of gene therapy and targeted drug delivery [12-24].

The purpose of this study was to explore the feasibility, safety, and efficacy of MRgHiFUS as a novel approach for renal sympathetic denervation in a swine model.

## Methods

### Animal study

The study was approved by the local animal research committee (Tierversuchs-Kommission des Landes Berlin (http://www.berlin.de/lageso/gesundheit/tierversuchskommission/index.html)). The domestic swine model was chosen for the study since its renal anatomy including arterial diameter and morphology is similar to that of humans [25]. Ten normotensive domestic pigs (weight, 27.2 ± 1.8 kg at baseline and 33.2 ± 7.1 kg at 4-week follow-up; average diameter of the renal arteries, 3.6 and 3.8 mm (l, r)) were used to examine the effect of MRgHiFUS therapy on the sympathetic renal nerves by assessing norepinephrine (NE) concentration in the renal parenchyma and histological changes in the renal sympathetic nerves. Ten pigs (nos. 1–10) were treated unilaterally on the right side with the contralateral nontreated kidney serving as control. Coincidentally, pig 7 had a congenital solitary right kidney. Therefore, the measured NE value was compared with the average of all NE values of the contralateral side of the other nine pigs. Since there was no appropriate acoustic window for the ultrasound beam due to a prominent transverse process, pig 10 received unilateral sham treatment on the right side (the same procedure as for regular treatment including the test sonication but not the therapeutic sonication) and therefore served as control to the other nine animals.

### MRgHiFUS procedure

Sonications were performed with a clinical MR imaging-integrated focused ultrasound system (ExAblate 2000; InSightec-TxSonics, Haifa, Israel).

A focused piezoelectric transducer array with a 120-mm diameter and operating frequencies between 1.0 and 1.5 MHz generated the ultrasound field. The array was located in the MR imaging table in a water tank (83 × 34 × 11 cm) containing degassed water and could be controlled electronically to modify the focal spot and the coagulated tissue volume. The pigs were positioned supine over the focused ultrasound device and fixed. To ensure optimal air-free acoustic coupling, a thin gel pad was placed on top of the positioner under the animal. Pretreatment imaging included acquisition of T2-weighted (T2-w) fast spin echo (FSE) sequences in three orthogonal planes. Posttreatment imaging immediately after the sonication procedure and 4 weeks after the intervention consisted of fat-saturated (fs) T2-w FSE sequences and a contrast-enhanced three-dimensional (3D) T2-weighted (T1-w) fast gradient echo (FGRE) MR angiography sequence (Table 1).

**Table 1:** MRI protocol for MRgHiFUS for renal sympathetic denervation

**Protocol**	**Sequence**
Preprocedural planning	T2-w FSE: axial, sagittal, coronal
	TR/TE 2,700/102 ms, ETL 20, bandwidth 15.6 kHz, FOV 36 cm, matrix 256 × 256, NEX 1.5, SL 4 mm
MRI temperature monitoring	Phase imaging with the use of a FSGRE sequence, TR/TE 26.4/13.1 ms, flip angle 30°, bandwidth 5.7 kHz, matrix 256 × 128, FOV 36 cm, SL 3–5 mm
Postinterventional control	Fat-saturated T2-w FSE TR/TE 2,700/101 ms, ETL 20, bandwidth 15.6 kHz, FOV 36 cm, matrix 256 × 256, NEX 1.5, SL 4 mm
MR angiography	T1-w contrast-enhanced MR angiography FGRE 3D, TR/TE 3.45/1.2 ms, SL 1 mm, imaging frequency 63.8 kHz, pixel bandwidth 420, FOV 36 cm, matrix 384 × 376, flip angle 25°

*TR* repetition time, *TE* echo time, *ETL* echo train length, *FOV* field of view, *NEX* number of excitations, *SL* slice thickness, *FSGRE* fast spoiled gradient-recalled echo.

After image transfer to the MRgHiFUS workstation, the target volume was defined.

In order to ensure and verify exact focusing of the ultrasound beam to the target region, calibration was performed prior to treatment. For calibration, in each pig, one to five test sonications were performed by focusing the ultrasound beam on the ipsilateral longissimus lumborum muscle. Once a temperature elevation was achieved at the targeted spot, monitored with real-time proton resonance frequency (PRF) MR thermometry, the ultrasound beam was focused at the renal periarterial tissue.

The levels of acoustic energy were chosen in accordance with the protocol established for the treatment of uterine fibroids in our institution. The maximum acoustic powers applied ranged from 100 to 166 W (average 133 W).

**Figure 1:**
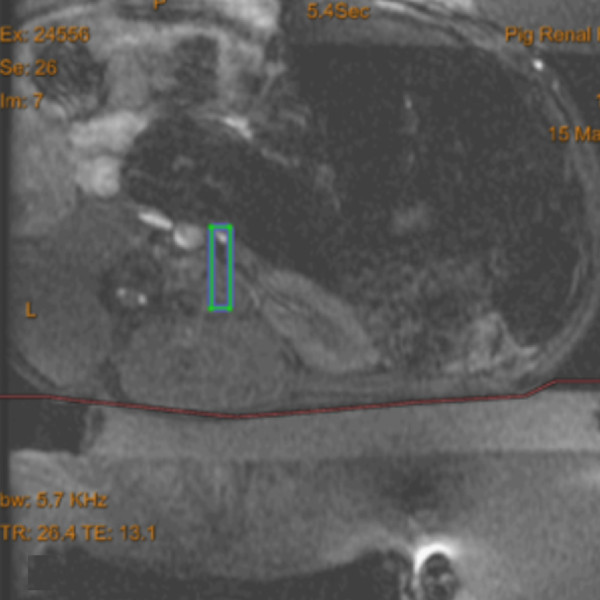
**Sonication planning image.** The sonication area lies within the *blue-green bar*, which includes the renal artery and the surrounding periarterial tissue.

For renal denervation, the first sonication was placed at the ostium of the right renal artery, and the following sonications in 5-mm overlapping intervals were around this spot (Figure 1). Sonication spots were chosen visually to completely cover the periarterial tissue. Further details are listed in Table 2.

**Table 2:** Details of the test and therapeutic sonications in pig nos. 1–9 (mean values ± standard deviation)

	**Test sonications**						**Therapeutic sonications**					
	**Number of sonications**	**Power (W)**	**Energy (J)**	**Duration (s)**	**Spot length (mm)**	**Spot diameter (mm)**	**Number of sonications**	**Power (W)**	**Energy (J)**	**Duration (s)**	**Spot length (mm)**	**Spot diameter (mm)**
Pig1	3	26.3	525.3	20	17	4.1	15	100.4	2,291.3	22	17	4.1
Pig 2	5	25.8	514.8	20	17	4.1	10	141.7	3,208	22.8	28.2	6.6
Pig 3	2	39	465	12	24	4.9	8	146.6	1,759.8	12	25.1	4.8
Pig 4	2	33	319	12	24	5.6	12	136.8	2,355.5	17.2	27.8	5.8
Pig 5	2	30	599.5	20	26	6.4	7	165.6	3,309.3	20	28.9	7.2
Pig 6	1	27	542	20	26	6.4	7	141.6	2,827.1	20	26.9	6.6
Pig 7	2	25.5	507.5	20	26	6.4	9	103.3	2,578.8	25	23.8	6.4
Pig 8	2	30.5	614.5	20	27	6.4	9	113.3	2,755	23.9	27.5	6.2
Pig 9	3	23	463.3	20	27	6.4	11	147.2	2,947.3	20	27.1	6.7
Mean	2.4	28.9	505.7	18.2	23.8	5.6	9.8	132.9	2,670.2	20.3	25.8	6.0
SD	1.1	4.9	4.9	3.5	4.0	1.0	2.6	22.2	485.5	3.9	3.7	1.0

### Assessment of technical outcome

Technical success was assumed if the postinterventional T2-w images showed a signal increase in the renal periarterial tissue as a sign of edema, assumed to develop in response to thermal injury.

Procedure time was defined as the interval from the initiation of the localizing sequence to the end of the posttreatment T2-w FSE and T1-w MR angiography sequences. The time required for preparing the pigs including anesthesia and positioning on the MRgHiFUS table was not included.

### Assessment of safety and efficacy

The first two pigs treated were sacrificed 2 h after the intervention to assess immediate effects of focused ultrasound on vessels and perivascular tissues. The remaining eight pigs underwent follow-up MRI at 28 days and were euthanized in deep anesthesia immediately thereafter. Apart from the fat-saturated T2-w FSE sequences, the scan protocol included contrast-enhanced MR angiography and MR urography for the identification of ureteral stenosis, thrombosis, hydronephrosis, or other inadvertent damage to the nearby abdominal organs.

For histological analysis after nephrectomy, a tissue block which included the renal vessels and any associated soft tissues was dissected and immersed in 4% neutral buffered formalin. The renal artery and perirenal arterial stroma containing renal nerves were sectioned transversely at 3–4-mm intervals. Tissue samples were then sectioned into 5-μm slices and stained with a hematoxylin and eosin (H&E) stain and with an Elastica van Giesson (EvG) stain. An independent, experienced pathologist examined all histological slides, looking for any evidence of injury to the renal arteries and the periarterial renal connective tissue containing the renal nerves.

Furthermore, slices of renal parenchyma, adrenal glands, and periarterial connective tissue stroma containing the ureter, renal vein, and renal lymph nodes were examined for undesired effects such as thromboemboli, infarcts, and inflammation.

The NE concentration in the renal parenchyma was determined to assess the effectiveness of percutaneous renal sympathicolysis. Immediately after explantation, both kidneys were homogenized separately in 1.0% formic acid and centrifuged. NE measurement was performed following liquid extraction by high-performance liquid chromatography (HPLC) according to Bauch et al. [26]. Details of the method have been described elsewhere [27].

Blood pressure was measured in all pigs immediately before the intervention (baseline), directly after the intervention, and at 4-week follow-up.

## Results and discussion

### Technical outcome

Image quality was sufficient in all animals and allowed identification of the renal vessels and possible sonication spots. In all pigs, the test sonications in the nearby ipsilateral longissimus muscle led to an increase in temperature and change in MRI appearance with a focal increase in T2 signal intensity consistent with edema. An increase in temperature in the renal perivascular tissue could not be measured reliably due to the surrounding fat tissue. The mean duration of the procedure was 90.4 (±20.8) min. For denervation, a mean of 9.8 (**±**2.6) sonications with delivery of a mean acoustic energy of 2,670.2 J (±485.5) were applied to the perivascular renal tissue on the right side.

### Safety and efficacy

The procedure was well tolerated by all pigs. Major complications did not occur. A mild focal skin burn at the entry spot of the ultrasound beam occurred in three pigs. The results are summarized in Table 3.

**Table 3:** Overview of results with regard to MR signal intensity, histology, norepinephrine concentrations, and blood pressure

	**Signal increase in T2-w after intervention**			**Histological changes**	**Norepinephrine levels**^**a**^			**Blood pressure (systolic/diastolic)**		
	**No changes**	**Periosseous/transverse process**	**Perivascular tissue**		**Right**	**Left**	**Change in%**	**Before**	**After**	**4-week follow-up**
Pig 1		X			*285.6*	396.2	-27.9	145/46	*171*/100	X
Pig 2		X			*496.3*	577.1	-14	119/52	*162*/83	X
Pig 3		X			478.7	433.1	10	*132*/74	122/48	93/46
Pig 4		X	X	X	*445.4*	509	-12.5	139/84	*153*/88	135/83
Pig 5		X			*317.7*	346.1	-8.2	*130*/42	118/55	118/49
Pig 6		X	X		*309.1*	359.7	-14	145/39	*166*/33	130/48
Pig 7		X	X		*267.9*	429.5	-38	117/39	*144*/60	111/34
Pig 8		X			348.9	270.5	29	112/62	*152*/74	122/52
Pig 9		X			*575.8*	658.5	-12.6	75/24	123/50	*158*/66
Pig 10	X				494.4	295.8	67.1	102/54	85/29	*123*/45
Mean					391.7	442.2		124/51	146/66	124/54
Pigs 1–9					(±109.5)	(±121.1)				
Mean					402	427.6		122/52	140/62	124/53
Pigs 1–10					(±108.3)	(±123.2)				

^a^LLOQ 20 ng/g tissue. Pig nos. 1 and 2 were sacrificed 2 h after the intervention to assess immediate effects on vessels and perivascular tissues. The other eight pigs were euthanized after 4 weeks. T2 signal intensity increase, histological changes, NE levels of the treated right (italicized) and untreated left kidneys (ng/g) at 4 weeks, and blood pressure values (systolic/diastolic in mmHg) before and after the intervention is given. All treated pigs developed edema in the tissue adjacent to the transverse process. Edema in the periarterial tissue was found in three pigs and corresponding histological changes in one pig. NE was lower on the treated side in seven pigs (italicized). Two pigs and the sham-treated pig had higher NE levels on the treated side. Average systolic and diastolic blood pressure 4 weeks after the intervention did not differ significantly from baseline.

### MRI visualization of thermal injury

**Figure 2:**
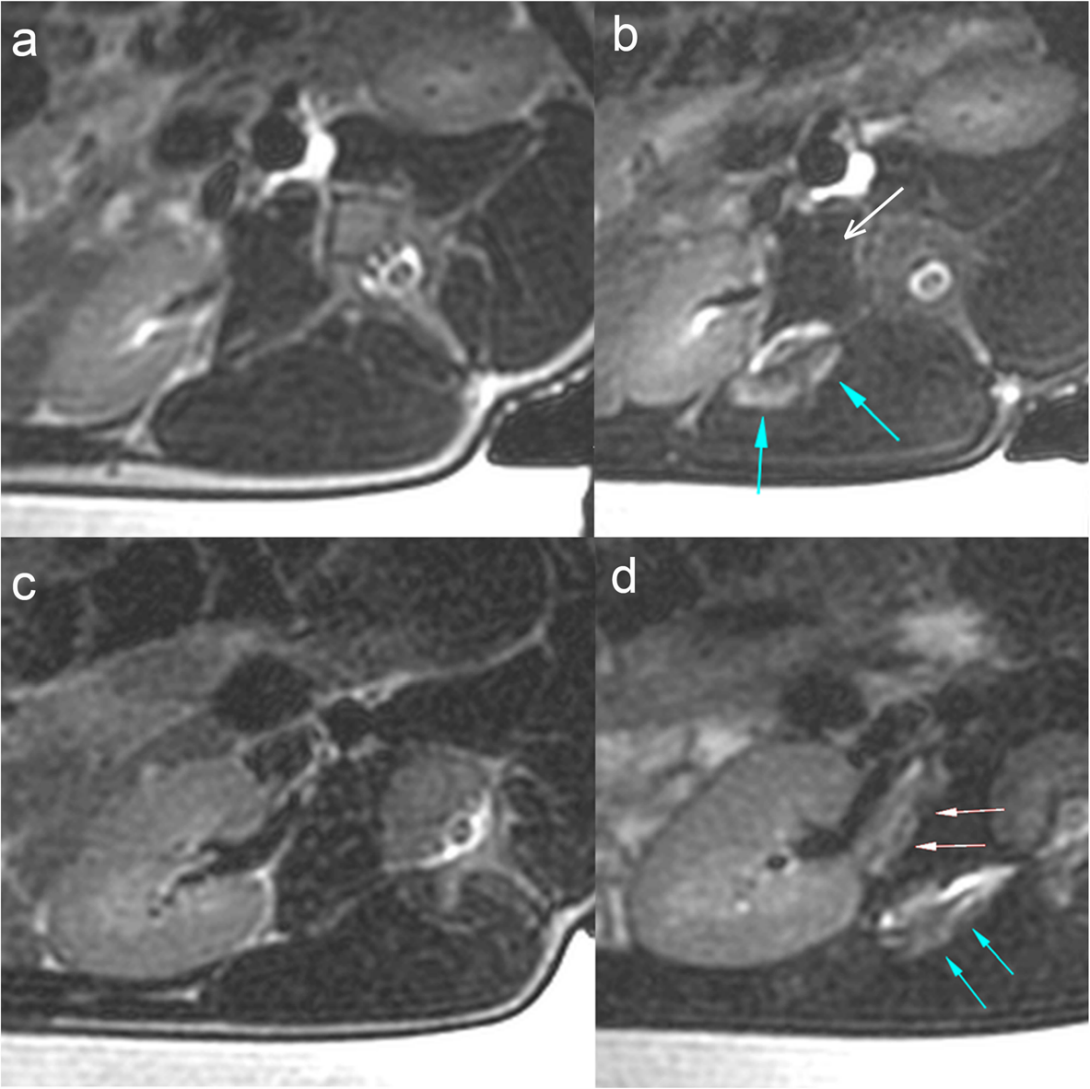
**T2-w FSE images before and after the intervention (pigs 5 and 4). (a, b)** T2-w FSE images before and after the intervention (pig 5). Postinterventional T2-w images show a signal increase within the paravertebral muscle adjacent to the transverse process (*blue arrows*). No signal increase is seen in the region of the perivascular tissue (*white arrow*). **(c, d)** T2-w FSE images before and after the intervention (pig 4). Postinterventional T2-w images show a signal intensity increase adjacent to the transverse process (*blue arrows*) and in the periarterial tissue (*white arrows*).

After the interventions, an increase in signal intensity in the T2-w MR images due to edema caused by tissue degeneration was found in the paravertebral muscle adjacent to the transverse process of the lumbar vertebral body in all pigs except for the sham-treated pig (Figure 2a,b). A similar signal increase was found in the perivascular tissue of three pigs (Figure 2c,d). In nine of the ten treated animals, temperature maps during the intervention revealed a significant, almost linear temperature elevation in the muscle around the transverse process and lateral to it. Reliable temperature measurement in the target area of the periarterial tissue was not possible. MRI and MR angiography 4 weeks after the intervention showed a normal right renal artery with no sign of stenosis or other complications of the surrounding tissues/organs.

### Histopathologic assessment

**Figure 3:**
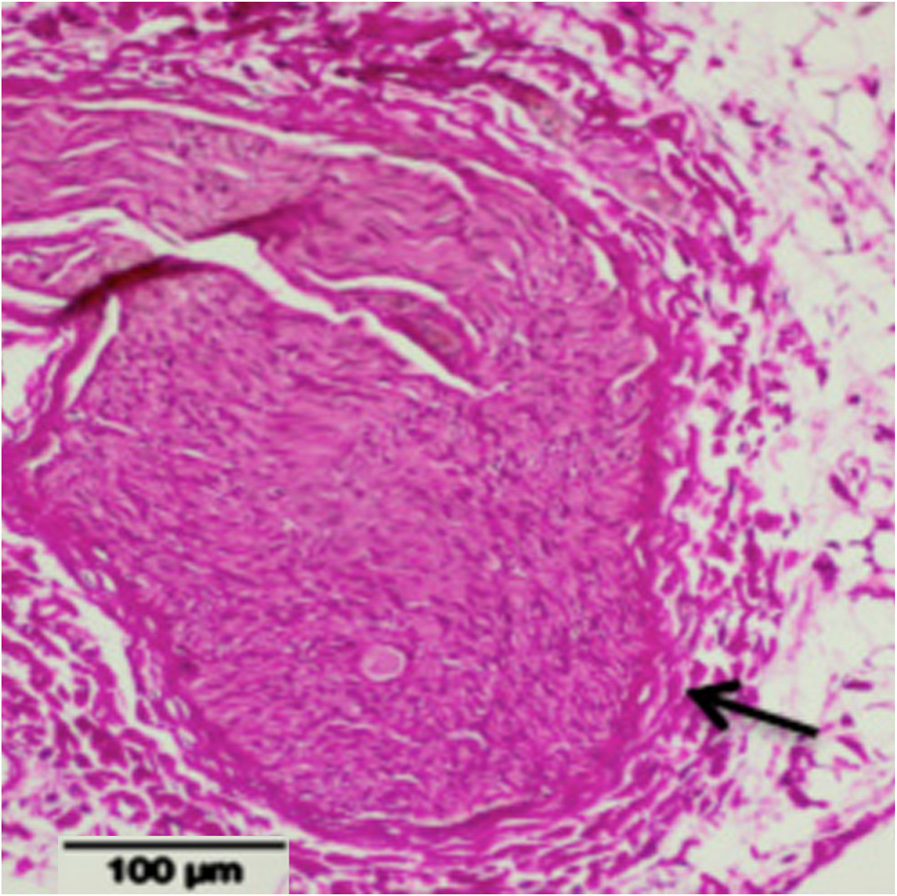
**Magnified view of a degenerated sympathetic nerve fiber (pig 4).** Degeneration is indicated by a thickened perineurium (*black arrow*) as a correlate of fibrotic remodeling (Elastica van Gieson stain).

In none of the nine treated pigs did histopathologic analysis reveal significant signs of neural injury or degeneration of the perivascular renal tissue on the treated side.Only in one pig (pig 4), where MRI showed signal alterations, were slight changes consistent with degeneration found in perivascular structures (Figure 3).

### Norepinephrine measurement

In the first two pigs as well as in five of the seven pigs with 4-week follow-up, renal NE was lower on the treated side with the difference ranging from -8.2% to -27.9% (the calculated difference in pig 7 was -38%). In two pigs, NE was higher (+10% and +29%) on the treated side. On average, there was no significant difference (*p* = 0.078, paired *t* test) in NE between the treated right (391.7 ± 109.5 ng/g) and untreated left (442.2 ± 121.1 ng/g) sides. In pig no. 10, sham treatment was associated with a significantly lower NE level on the untreated side (295.8 vs. 494.4 ng/g) (Table 3).In one pig (no. 4), treatment led to an NE decrease of -12% combined with visual changes in MR signal intensity corresponding to edema of the renal perivascular tissue and the region close to the transverse process. Moreover, histopathologic analysis revealed signs of degeneration of sympathetic nerve fibers (Figure 3).

In two pigs, treatment led to MR signal alterations in the perivascular tissues and a decrease in NE but was not accompanied by histological changes in the sympathetic nerves. In the remaining pigs, edema was only found in the tissue surrounding the transverse process. In this subset, NE levels were variable with some pigs even showing lower values on the untreated side.

### Blood pressure

Pre- and postinterventional systolic and diastolic blood pressure did not differ significantly with average values of 124/51 mmHg before the interventions and 124/54 mmHg at 4-week follow-up. Compared to baseline, seven of nine pigs showed increased systolic and diastolic blood pressures immediately after the interventions with average values of 140/62 mmHg.

Despite initial success in treating resistant hypertension, endovascular RFA of renal sympathetic nerves has procedural limitations. Not all patients are candidates for endovascular RFA since there are certain anatomic requirements to be eligible for the procedure. Furthermore, clinical outcomes are variable with reported rates of nonresponders ranging from 0% to 31% after 1 month and from 0% to 26% after 6 months [28,5-32]. Since the recently published results of the HTN-3 trial show no significant difference in blood pressure reduction after 6 months between patients treated with RFA-based renal denervation and sham-treated patients [1], it is important to investigate possible alternative methods for renal denervation.

MRgHiFUS has the potential for noninvasive renal sympathetic nerve ablation because the focused ultrasound beam can produce a precise focal temperature increase and thus induce circumscribed tissue degeneration. With its high intrinsic contrast, MRI can visualize and clearly define the target area for sympathetic denervation. MRgHiFUS is potentially safer for patients, as it is noninvasive and therefore less susceptible to complications. Furthermore, there are no anatomical restrictions in terms of renal artery diameter and length.

To our knowledge, this is the first study investigating the feasibility of MRgHiFUS for renal sympathetic denervation in a porcine model.

As to side effects, there were neither major complications nor any inadvertent perirenal damage. The mild focal skin burns seen in three pigs can be explained by incomplete removal of the pigs’ bristles, leading to insufficient coupling between the transducer and the skin and therefore a large change in impedance causing local heating. Since human hair is much thinner and easier to remove, this should be no matter of concern.

Regarding the efficacy of this technique, the results of this initial study indicate that renal sympathetic denervation with MRgFUS in pigs is difficult and leaves much to be desired in terms of efficacy.

Wang et al. investigated renal denervation with HiFUS in canines using color Doppler flow imaging (CDFI) for guidance [33]. In this study, up to six ablations at three foci of the renal artery led to a significant decrease in plasma NE concentration and in systolic and diastolic blood pressure on days 6 and 28 after the interventions and to degeneration of the targeted nerve fibers.

**Figure 4:**
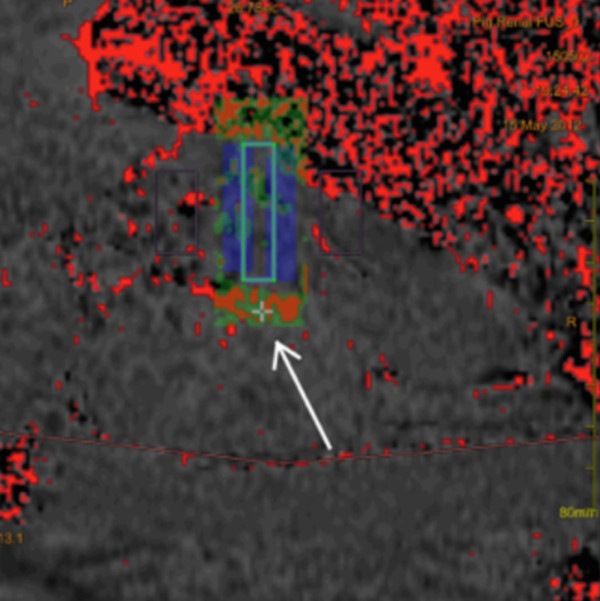
**Real-time temperature map with color-coded visualization of different tissue temperatures ( *blue *: low temperature/ *red *: high temperature).** The temperature map image shows focal heating (*white arrow*) in the dorsal periarterial space, possibly corresponding to energy absorption at a longish structure of thickened tissue consistent with an adjacent fascia. The bar indicated the region of sonciation.

The lower effectiveness with regard to NE decrease, changes in MRI signal intensity, and histological changes in our study is most likely related to an insufficient energy deposition caused by a small acoustic window with beam path impediment in the porcine model. In HiFUS, orthogonal beam orientation to the target region and an unimpeded beam path are crucial for minimizing energy reflection and absorption [24]. Bones or fascia within the acoustic window may distort and partially reflect the beam and may absorb a large amount of the applied ultrasound energy. In previous studies investigating the treatment of uterine fibroids, MR thermometry showed an inhomogeneous energy deposition within the target region (i.e., focal hot spots), which were primarily attributable to ultrasound beam distortions induced by the overlying tissue layers [34,35]. These effects can cause focal hot spots behind the absorbing structures instead of the target region [24].In our experiments, MR temperature maps showed a linear temperature increase in the dorsal perirenal space (Figure 4) in some pigs. This heating pattern might be explained by energy absorption of the prominent transverse process and its adjacent fascia. Postinterventional T2-w images confirmed edema in the tissue adjacent to the transverse process in most pigs, caused by thermal injury.

The prominent transverse process in pigs limited the acoustic window for sonication of the right renal artery. To maximize the acoustic window, the pigs were placed in a right oblique position. As a result, however, the ultrasound beam was suboptimally angulated and had to partially pass through the right kidney in most pigs, resulting in a further loss of energy.

Since all test sonications led to heat-induced tissue degeneration in the longissimus muscle, our results indicate that, as a result of the absorption effects described above, the therapeutic sonication beam was too weak to induce sufficient thermal ablation of the perivascular sympathetic fibers.

In our series, pig 5 was treated with the highest acoustic power level (166 W) and no thermal effects were shown. On the other hand, pig 7, which was treated with the second lowest power level, showed signs of denervation. This disparity between power level and therapeutic effect indicates that the chosen energy levels might have been sufficient for renal denervation had the effects of energy absorption not been so substantial.

In humans, the acoustic window is presumed to be larger due to a greater distance between the kidneys and the relatively smaller transverse processes. Furthermore, with increasing distance between the ultrasound transducer and the target region, the cone-shaped ultrasound beam is increasingly straightened. A straighter beam in turn reduces the number of affected tissues, resulting in fewer reflections.

Precise monitoring of the temperature and temperature increase in the treated tissue was difficult. PRF thermometry is a method for calculating the applied thermal dose and determining tissue damage during sonication using temperature-dependent phase changes in gradient-recalled echo (GRE) pulse sequences [36-38]. While working well in fibrous soft tissue or parenchymal soft tissue, this method is limited in fat as the phase dependence of lipids is almost independent of temperature [39,24]. Since other techniques of temperature mapping in the periarterial tissue, which consists mainly of fat, were not supported by our MRgHiFUS device, reliable information on energy deposition and the resulting tissue degeneration around the renal artery could not be obtained. A reliable method of temperature monitoring in fat would help in identifying insufficient heat development in the target region and enable adjustment and optimization of the sonication parameters during the intervention.

Since all pigs had normal blood pressure values before the intervention, a drop of systolic and diastolic blood pressure was not expected. The increase in systolic blood pressure, observed in six pigs immediately after the interventions, is likely caused by interventional stress.

This study has some limitations. All pigs were healthy and normotensive. Results may be different in a hypertension model. *In vivo* determination of NE spillover to characterize sympathetic activity was not available. Therefore, we measured NE concentration in the renal parenchyma, which has shown to be a good surrogate parameter in earlier studies [27]. Interestingly, in few pigs, NE of the treated kidney was higher than that of the nontreated control. Furthermore, NE values differed between both kidneys in the sham-treated pig. This finding corresponds to the results of NE measurements in a series of naive pigs of the same age and species, in which the NE levels of both kidneys also varied by up to 35% (Ulrich Speck, unpublished data). With a mean NE level and standard deviation (SD) of 391.7 ± 109.5 to 427.6 ± 123.2, our results are similar to the values of this ‘control’ group (333.4 ± 50.5 ng/g). Apart from random variation, potential causes for higher NE and SD values in the treated kidneys may be attributable to variations in the time span between euthanasia and kidney homogenization and storage of the homogenates.

In conclusion, this feasibility study shows that MRgHiFUS can induce thermal effects to the renal periarterial sympathetic nerves. Yet, the treatment did not result in significant renal sympathetic denervation in a pig model. This is likely due to insufficient periarterial energy deposition, which in turn was mainly attributable to energy absorption by the transverse processes and the fascia, which both lie in the beam path. Since MRgHiFUS treatment in humans is expected to be easier to perform, due to easier access to the renal sympathetic nerves, more experimental studies are needed to further investigate the therapeutic potential of MRgHiFUS for renal denervation.

## Competing interests

The authors declare that they have no competing interests.

## Authors’ contributions

The concept and design, acquisition of data, analysis and interpretation of data described in this study were carried out by PF and FS. PF prepared the initial draft, which was revised by FS, RG and BH. LH, AB, NS, JS and JK participated in the data acquisition and analysis. All authors read and approved the final manuscript.
